# Mathematical model of the feedback between global supply chain disruption and COVID-19 dynamics

**DOI:** 10.1038/s41598-021-94619-1

**Published:** 2021-07-29

**Authors:** Xingyu Li, Amin Ghadami, John M. Drake, Pejman Rohani, Bogdan I. Epureanu

**Affiliations:** 1 Department of Mechanical Engineering, University of Michigan, Ann Arbor, USA; 2 Odum School of Ecology, University of Georgia, Athens, USA; 3 Center for the Ecology of Infectious Diseases, University of Georgia, Athens, USA; 4 Department of Infectious Diseases, University of Georgia, Athens, USA

**Keywords:** Infectious diseases, Viral infection, Information technology, Health care economics, Scientific data

## Abstract

The pandemic of COVID-19 has become one of the greatest threats to human health, causing severe disruptions in the global supply chain, and compromising health care delivery worldwide. Although government authorities sought to contain the spread of SARS-CoV-2, by restricting travel and in-person activities, failure to deploy time-sensitive strategies in ramping-up of critical resource production exacerbated the outbreak. Here, we developed a mathematical model to analyze the effects of the interaction between supply chain disruption and infectious disease dynamics using coupled production and disease networks built on global data. Analysis of the supply chain model suggests that time-sensitive containment strategies could be created to balance objectives in pandemic control and economic losses, leading to a spatiotemporal separation of infection peaks that alleviates the societal impact of the disease. A lean resource allocation strategy can reduce the impact of supply chain shortages from 11.91 to 1.11% in North America. Our model highlights the importance of cross-sectoral coordination and region-wise collaboration to optimally contain a pandemic and provides a framework that could advance the containment and model-based decision making for future pandemics.

## Introduction

Severe Acute Respiratory Syndrome Coronavirus 2 (SARS-CoV-2) is a virus that infects the upper and lower respiratory tract^1,2^ and is the causal agent of the Coronavirus Disease 2019 (COVID-19). In 2020, SARS-CoV-2 affected over 200 countries and territories and caused 117.8 million infections and 2.6 million fatalities after being declared a pandemic by World Health Organization (WHO). Despite a century of successful prevention and control efforts, infectious diseases remain an important global problem in public health and socioeconomic disruptions. COVID-19 is not the first virus that led to the global pandemic. Many people have drawn comparisons between COVID-19 and pandemics of influenza that occurred in 1918, 1957–1958, 1968, and 2009^3^. The scientific consensus is that infectious disease emergence has accelerated in recent decades while the current ability to anticipate the next pandemic remains very limited^4^. To address challenges and risks in future pandemics, the dynamic interconnection between managerial decisions, infectious diseases, and shortages of the health care equipment must be considered.

The current global economic situation and ongoing recession were triggered by the COVID-19 pandemic, in part due to supply chain disruptions. Thus, there is a feedback such that infectious disease outbreaks not only deeply affect the world economy and global health, but also fundamentally change society’s ability to contain the outbreak. During COVID-19, major enterprises owning more than 12,000 production facilities were shut-down due to quarantine policies, which lead to severe supply chain disruptions^5,6^ and a huge dip in international trade, which declined between 13 and 32%^7^. The shortage of critical health care resources due to supply chain disruptions and difficulties in international trade then had a significant impact on timely delivery of health care service^8,9^. Since the beginning of the pandemic, efforts by local health departments all over the world have sought to optimize response strategies to mitigate the outbreak. These efforts, however, have been halted by limited critical health care resources at different times and places^10^. The shortage of health care resources originates from surging demands caused by accelerating numbers of COVID-19 cases, misinformation, panic buying, and stockpiling^11^. Resource shortages not only raise health risks but also accelerate the spread of the disease, which exacerbates resource shortages in a vicious circle. With the accelerated spread of the disease, there is a pressing need to sustain the supply chain of critical items to prevent the detrimental feedback between the supply chain disruption and disease growth. This study fills this research gap by applying ecological and managerial knowledge to develop a tool that explicitly captures positive feedback in supply chain disruptions and infectious disease dynamics.

COVID-19 has brought a worst-case scenario affecting critical stock availability: a rapid surge in demand combined with the shortage of substantial raw materials due to global supply chain disruptions^12^. Such a disruption in the supply chain network can also limit the development and delivery of vaccines^13^. To mitigate resource shortages, simply demanding more critical medical supplies is not enough. There are only a few companies that have the expertise to manufacture these products and are able to ramp up production under global supply chain disruptions.For instance, sanitizer manufacturing companies in Australia started facing shortages of required raw materials and ingredients soon after increasing their capacity of producing sanitizing gel^12^. Ford’s effort of building 50,000 ventilators in July was delayed due to part shortages and challenges in scaling up the part production^14^. Avoiding such disruptions required that policymakers both guide these companies to maximize their production capacities and coordinate other industries into this effort to guarantee material supplies^15^. Advanced models and simulations must inform policymakers.

This pandemic revealed that even high income countries compromised the delivery of health care resources and services as a result of rapid spread and depletion of material stocks. Studies show that the shortage of health care resources in the USA, especially N95 masks, was predictable and preventable with publicly available supply chain data and a careful management strategy^16^. Worldwide efforts to mitigate COVID-19 revealed the necessity of an data-driven model to represent changes in the evolving pandemic, which fuses the diverse data sources including required quantity, rate of use of health care resources and global supply chain data^17^. As the virus’s global spread escalated, demand for surgical masks skyrocketed in China, beginning in February 2020. As the largest mask exporting country, China paused its export of face masks^18^ and began importing from Europe, Japan, and the United States to alleviate mask shortages^19^. Awareness of such surge demands of health care resources has long-term impacts on managerial decisions and disease control. However, the growth of the epidemic creates even more turbulent supply chain and disease situations. It is challenging to foresee intuitively the outcomes from the complex interconnections in order to wisely distribute resources in the global supply chain.

We propose that the development of the Internet of Things (IoT) has created the conditions for the digitalization of supply chains, providing the essential data for addressing challenges during pandemics^20^. Smart sensors and communication technologies are becoming more practical, which connects the global manufacturing assets, i.e., robots and machines, as a digital supply chain network^21^. Digital supply chain connects the physical process, including, manufacturing process and supply chain activities to the virtual models, also known as a digital twin^22^. The data and decisions simulated and optimized in the digital twin can be used to improve the performance of physical processes. Further, digitalization enables the supply chain to evolve over time adapting to disruption risks of manufacturers and changes in market environment, which substantially extends the mitigation capabilities of supply chain disruptions^23^.

Here we report on a model coupling disease dynamics and the digital global supply chain network to understand the essential feedback between economic productivity and public health. Our approach makes use of real-world input–output trade data and ongoing disease data to represent the real-time risks and impacts of an infectious disease. A network proactive control (NPC) is formulated to create an agile and time-sensitive containment strategy, which optimizes worldwide managerial decisions considering predictive impacts on a daily basis. Using data-based simulations, we first investigate how different managerial strategies affect the coupled economic-epidemic dynamics and then project it to disease impacts on public health and the global economy. This new approach can be used as a stress-test tool to evaluate the robustness and resilience of the supply chain in public health emergencies, enabling a holistic impact analysis of cross-industry coordination and inter-region collaboration to contain emerging diseases. The proposed approach of coupling the disease dynamic network and the supply chain network can be used for other future events similar to the COVID-19 pandemic.

## Model

### Model of supply chain disruption in a substantial demand change and quarantine policy

**Figure 1:**
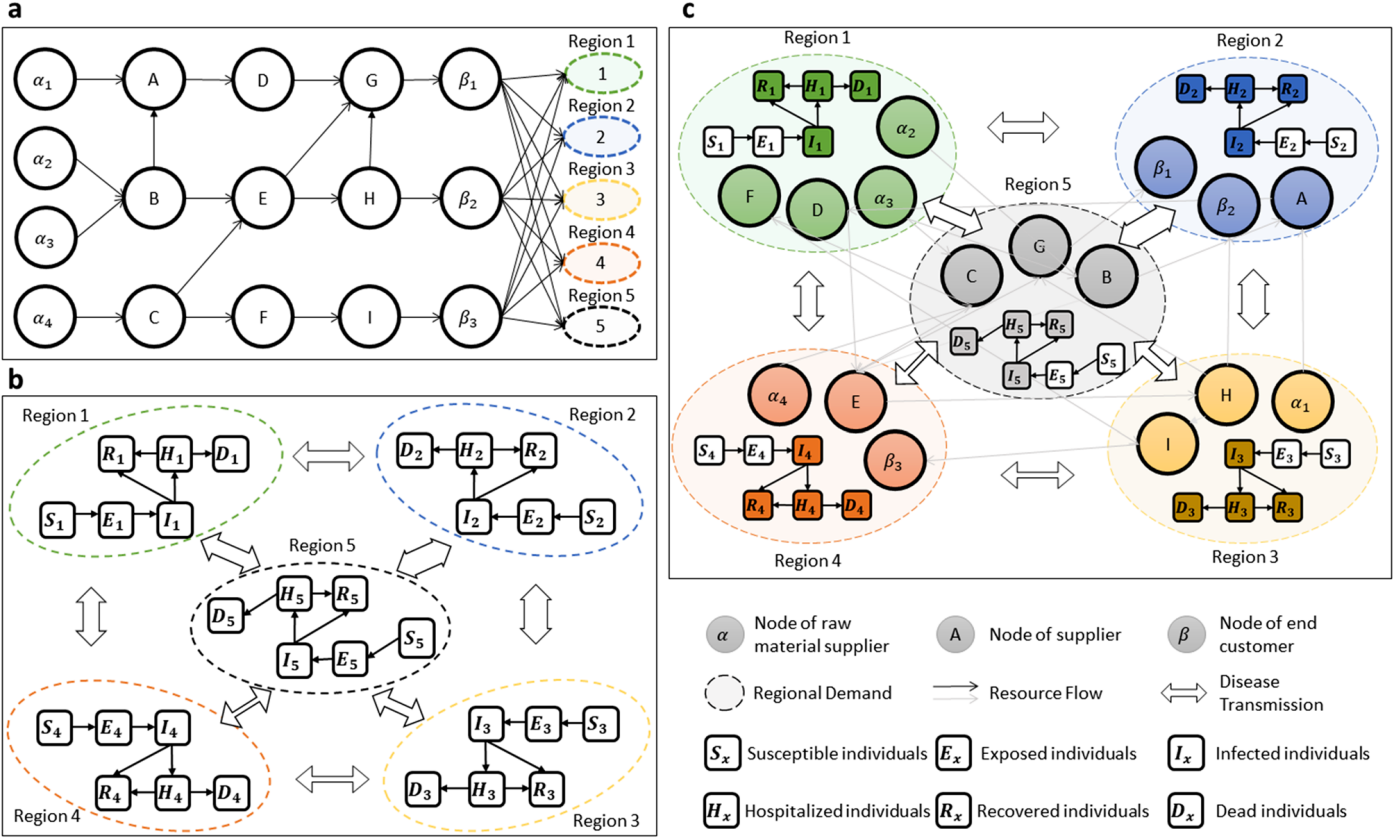
System Model of supply chain and disease dynamics: **(a)** a classic supply chain network with three types of products; **(b)** a classic multi-patch disease network for COVID-19; **(c)** the model of multi-patch disease and the production and supply chain.

Figure 1a shows a digital supply chain network including activities, people, entities, information, and resources across diverse industries. The arrow line represents the supplier-customer relationship between manufacturers in the supply chain. Figure 1c visualizes the model that accounts for feedback between shortages of health care resources, supply chain and local disease dynamics. Under pandemic conditions, production is disrupted due to restricted transportation, shortage of raw materials from suppliers caused by lockdown policies, and changes in demands, especially for critical health care products. Decision makers need to face local production disruptions while satisfying regional health care supply needs. Thus, regional disease dynamics are critical factors in making managerial decisions, i.e., dyed and reorganized nodes in Fig. 1c. Pandemic complicates challenges the supply chain management.

Demand for health care resources increased with the growth of pandemic^24^. For example, masks are a health care resources in medical procedures and also recommended by WHO to provide an adequate protection against COVID-19 in public activities^25^. In our model a disease-dependent demand function explicitly represents the surge in medical needs by correlating the demand with the number of infected and hospitalized individuals. In addition, the disease has a substantial impact by disrupting the production capacity for supplies due to quarantined labor force^26^ and constrained factory working hour^27^. Studies have found the optimal lockdown policy to be dependent on the prevalence of disease in the local population^28^. We also assume the optimal policy to be adopted, in which case we use a disease-dependent capacity function to connect production capacity to the current disease situation.

### Model of disease dynamics during the shortage of medical supplies

Figure 1b shows a multi-patch (metapopulation) compartmental model^29^ that represents the kinetics of an epidemic in an idealized susceptible population of connected regions. Studies indicate that the proper usage of face masks can synergistically act together with social distancing to reduce the virus transmission rate^30^. In addition, reliable access to health care resources, including medical supplies, devices, and therapy, is critical for hospitals to continue to function adequately^17^ and to deliver stable and adequate health care^31^.

A shortage-dependent transmission function in the model calculates the transmission rate in each region according to the amount of health care resource shortage per capita. Items like masks and face shields are critical items for preventing COVID-19 as suggested by the WHO^32^. The unsatisfied demands of health care resources impact the ability to adhere to ideal infection control, health worker safety and treatment^33–35^ thus changing the transmission of the disease. In COVID-19, special medical devices like ventilators have been required. One ventilator per patient is not possible due to the shortages of ventilators in many countries, which increases the death rate in the population^36,37^. Our model includes a shortage-dependent fatality function that measures the death rate and recovery rate as functions of timely availability of supplies of health care resources.

## Results

Considering the geographical separation and economical connections, five regions are selected and connected as a network to represent the world-wide pandemic and global supply chain, i.e., Asia, European Union (including United Kingdom), Latin America, North America, and Oceania. We aggregated all manufacturers that produce goods and services in each region, and considered the capability of trading among regions. Three data sets are used for estimating model parameters. In the disease network, the transmission rate is estimated on a daily basis by fitting pandemic data reported by Johns Hopkins University Center^38^. Tourism data provided by the World Tourism Organization^39^ and international air travels history reported by the International Air Transport Association (IATA) are aggregated to approximate the connectivity among regions. A digital supply chain network is built from the Global Trade Analysis Project (GTAP) database (version 10)^40^. Industries are grouped into 10 sectors defined by GTAP. The human health and social work (HHS) sector are separated as a special sector to represent the expenditure on health care resources in human health and social work^41^. Resources required by HHS are considered to correlate with the emergence of COVID-19^42^, reflecting the surge in demands of critical medical supplies. Assuming each regional sector as a producer, the following information is obtained for each regional sector: (1) bill of material in production, (2) pre-pandemic production capacity and (3) cross-regional resource flows at equilibrium.

**Figure 2:**
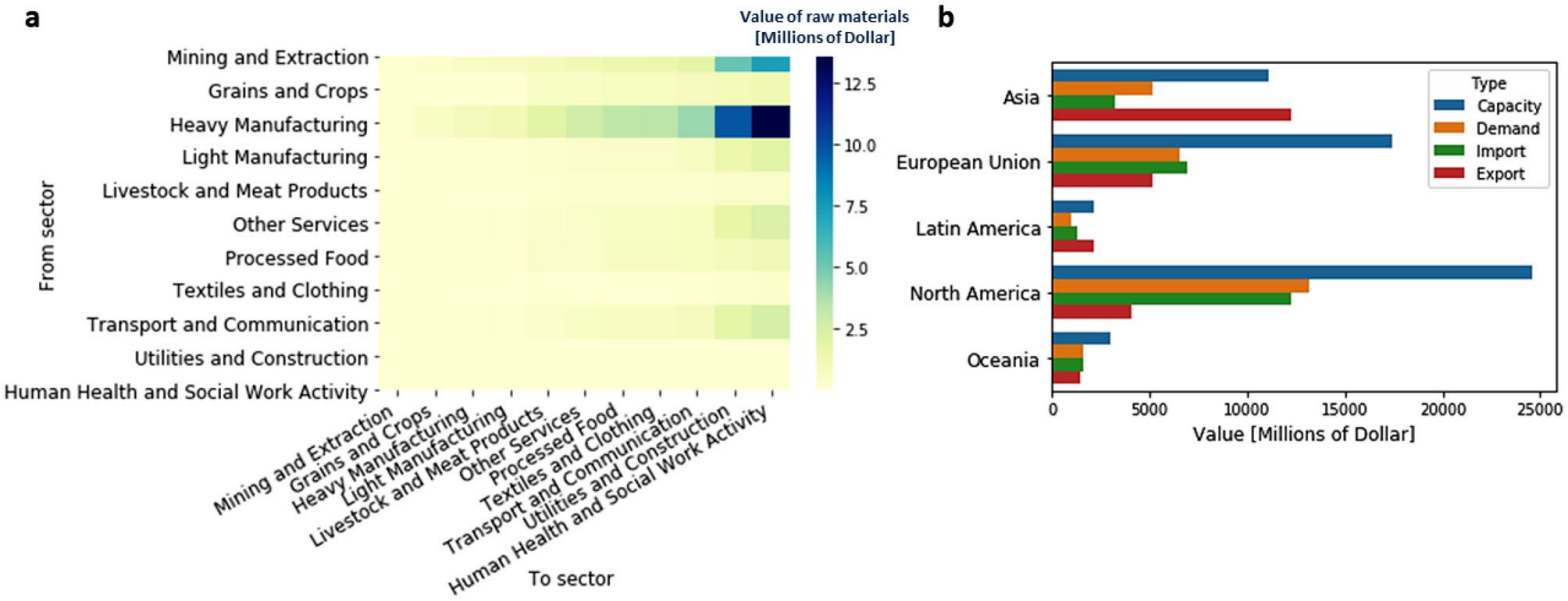
Global trade data analysis based on GTAP 10: **(a)** Values of required raw material from different sectors to satisfy the doubled production of each sector. The rows of the table define sectors that provide the raw materials, and the columns of the table define sectors that double their production. **(b)** Comparison of the production capacity, demands, and trade at the equilibrium between different regions. Here, the capacity is calculated by the total yearly production estimated from the total export and self-consumed resources. Demands are estimated based on the resource consumption from households and government. Trade history is based on resource exchanges in each region, including household, government, and companies.

Figure 2a lists all sectors used in this study, and compares the demand inputs from other sectors if productions of each sector were to double. Increasing HHS production depends on inputs from many other sectors. The major inputs are from heavy manufacturing, mining, and extraction. Light manufacturing, other services, and transport and communication are dependent sectors also. A strong dependence emphasizes the need for sectoral coordination to ensure sufficient material supplies are available to increase the production of health care resources. Figure 2b shows the diversity of production and demand among regions, in terms of capacity, demand, and trade at equilibrium measured in millions of dollars. North America has the highest HHS capacity, import, and demand, followed by the European Union. Latin America and Oceania have low production capacity and low needs for HHS resources. Asia is a major export region. Regional diversity highlights the need for analyzing the risks of regional production disruptions, as well as the customization of the containment strategy for each region.

### Cross-sector coordination

**Figure 3:**
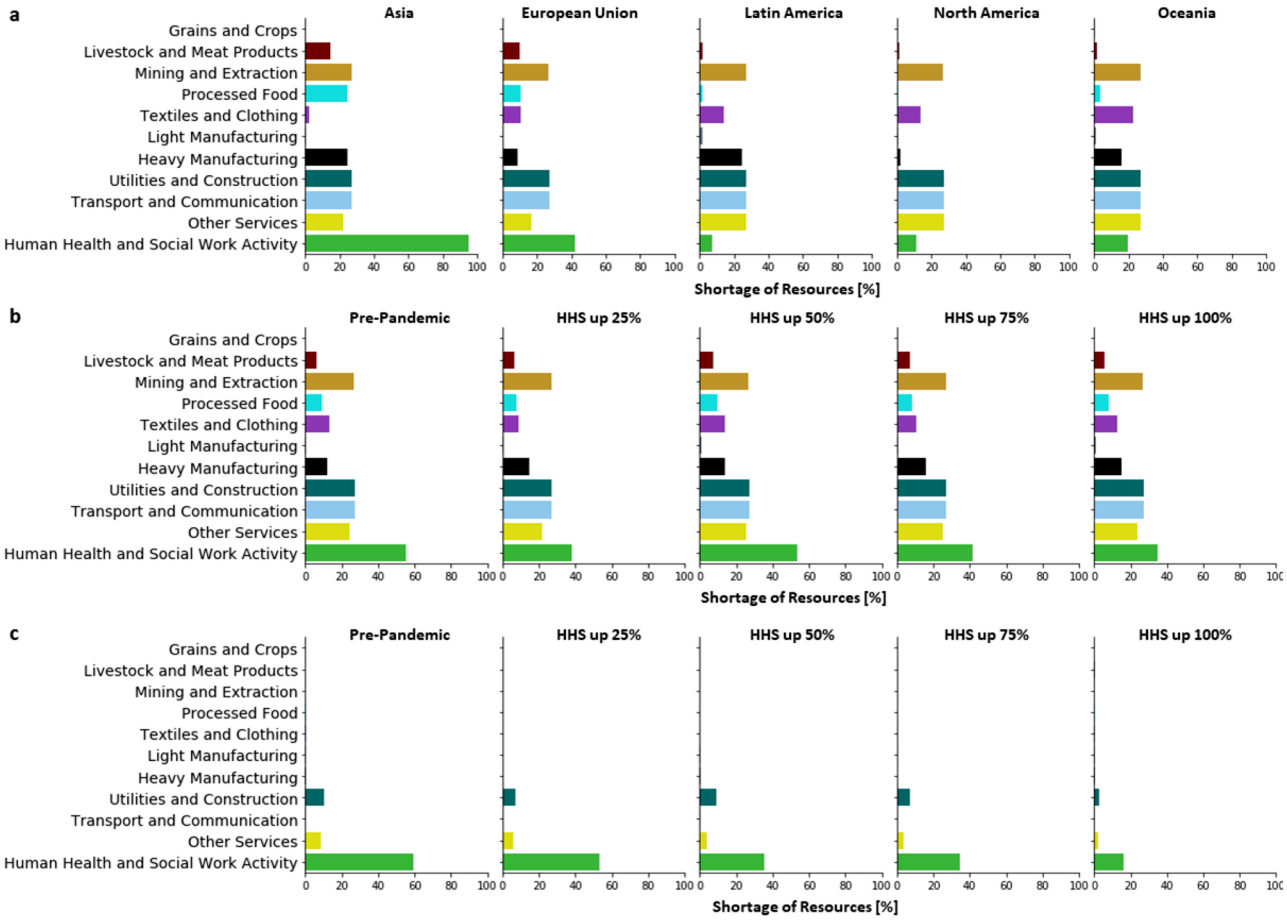
Sectoral impacts of increased HHS capacity. **(a)** Without coordinating other sectors, the regional shortage of resources after doubling the production of HHS in each region. **(b)** Without coordinating other sectors, the averaged shortage of resource in five ramp-up scenarios, in terms of the pre-pandemic capacity (100), ramp-up by 25% (125), ramp-up by 50% (150), ramp-up by 75% (175) and , ramp-up by 100% (200). **c)** With coordinated supply chains, the averaged shortage of resource in five production ramp-up scenarios.

We study positive feedback between disease dynamics and supply chain disruption in a 100-day simulation of the model initialized by COVID-19 data on March 1st 2020. Managerial decisions are updated daily by a proactive control of the supply chain network with consideration of production and inventory constraints. The model considers the impact of managerial decisions in terms of disease control and economic losses seeking a balanced solution. With a higher capacity of producing HHS resources, this containment strategy tends to raise the HHS production to alleviate the shortage of HHS resources so as to reduce the spread of the disease. Figure 3a shows the results of a stress test to identify the vulnerable sectors in satisfying the demands from a health emergency. Results show that Grains and Crops is the least impacted sector among all countries due to its weak dependence on other sectors. Transport and Communication is the bottleneck sector that limits further increase of HHS production. Because the differences in regional capacities, sectoral impacts also vary. Asia and Oceania are impacted the most in their Heavy Manufacturing sector. Interestingly, the analysis shows that the shortage of Processed Food, and Livestock and Meat Products in Asia are significantly greater compared to other regions, which is consistent with the raise in the price of pork reported in Asian countries^43,44^.

Figure 3b shows the shortages for four different HHS ramping-up scenarios. Despite the forced increase in capacity by the policy-maker, the capacity is dynamically updated according to the evolving disease situation by the disease-dependent capacity function. With increasing production capacity, the shortage of HHS does not reduce monotonically, but fluctuates around 40%. As a result, significant shortages are observed in other sectors, including Mining and Extraction, Transport and Communication, and Utilities and Construction, which overlap with dependent sectors in the Fig. 2a. Other sectors, such as Textiles and Clothing, suffer a remarkable shortage due to the shared input materials with the HHS resources, i.e., cloth and masks. Increase in the production capacity is not enough to meet the surge in demands due to the limitations of raw materials. Without coordinated sectors, ramp-up of production capacity incurs the overuse of materials in the existing stockpiles, which leads to further raw material shortage in the future, thus deepening the disruptions in producing HHS resources.

Figure 3c shows the sectoral impacts when sectors are coordinated, namely, all the other sectors are able to provide sufficient raw materials for HHS production. With increasing HHS production capacity, a clear decreasing trend of HHS shortage is observed, reducing from 60% to less than 20%. Note that even with sufficient production capacity, shortages exist in Other Services and Utility and Construction, which require raw materials from the HHS resources for production.

**Figure 4:**
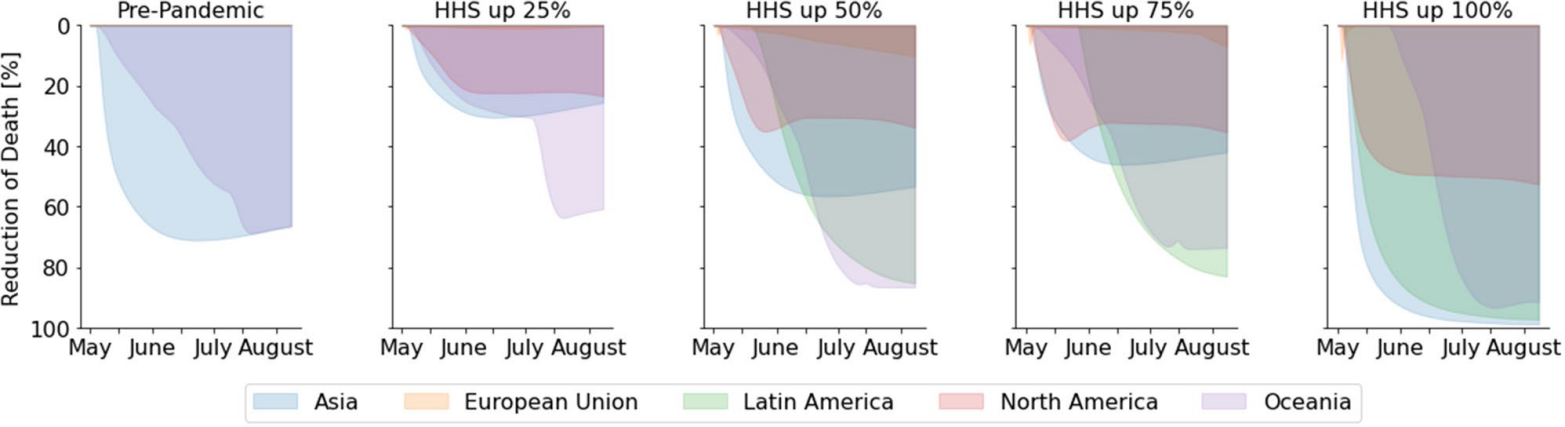
Disease impacts for increased HHS capacity. Assuming coordination of the supply chain is possible, the diagrams show the percentage of fatality reduction in different regions with the increasing production capacity of HHS resources, in terms of the pre-pandemic capacity (100), ramp-up by 25% (125), ramp-up by 50% (150), ramp-up by 75% (175) and , ramp-up by 100% (200). The fatalities simulated in the pre-pandemic capacities is used as a benchmark to calculate the reduction percentage.

Figure 4 shows the influence of improved HHS production on disease outcomes. As a benchmark, the number of fatalities is calculated based on simulation results according to the pre-epidemic production capacity. The percentage of reduction is calculated by comparing the total number of fatalities to the benchmark at each time instance. With coordinated supply chains, an increase in HHS capacity at early stage of pandemic substantially reduces the number of fatalities as well as the duration of the outbreak. A reduction in HHS shortage in the model reduces disease transmission rate, thus suppressing the number of newly added infections due to better personal protection. In addition, fatality rate decreases with more medical resupplies. As a result, the number of fatalities is reduced by 90% by doubling the capacity of HHS in March 2020.

### Lean resource management and regional collaboration

**Figure 5:**
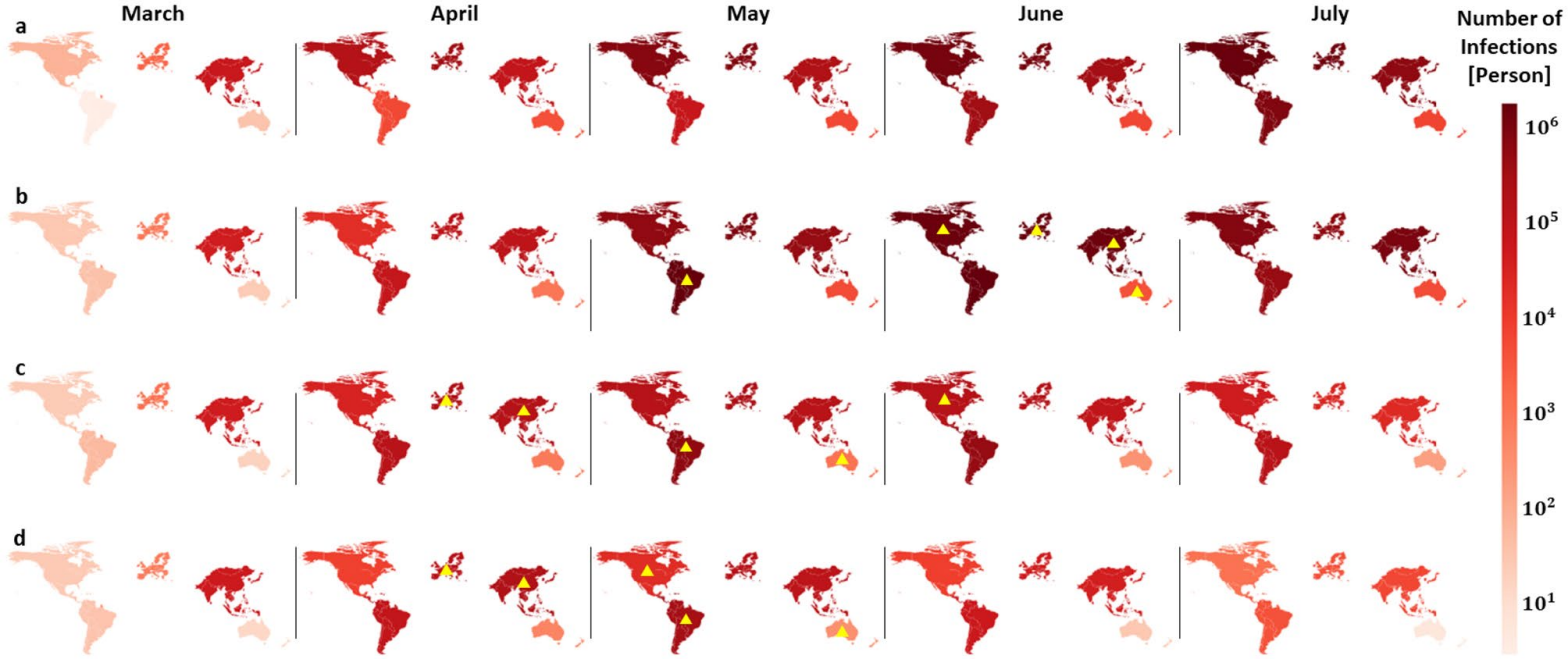
Impacts of strategies on infections. **(a)** Actual infections between March 2020 and July 2020 caused by the on-going COVID-19 pandemic. **(b)** Simulated infections using the PE containment strategy. **(c)** Simulated infections using the PE containment strategy. **(d)** Simulated infections using the OCN strategy. The color intensity in each region represents the number of infections. The yellow triangles mark the occurrence of a peak in infection.

We sought to confirm that key improvements are possible in lean management of HHS resources and regional collaboration by exploring the managerial decisions made in the model. Our analysis starts from March 1st 2020 based on COVID-19 data, and the model is simulated for 30 days based on equilibrium decisions. Then, a 100-day simulation is conducted based on three containment strategies: (1) pre-epidemic management strategy (PE): adopt the same managerial decisions as pre-pandemic; (2) optimization of digital supply chain strategy (ODS): adopt optimal digital supply chain managerial decisions considering the pandemic as a disturbance; (3) optimization of the coupled network model strategy (OCN): optimize the managerial decisions based on feedback in the model of coupled networks. Figure 5 shows the effectiveness of these containment strategies in terms of infections in different regions.

Figure 5a shows the actual number of infections during the COVID-19 pandemic, which is a benchmark for the proposed models. Figure 5b shows the number of infections in the PE strategy which skyrockets in the 3 months following March 2020. The magnitude of infections is similar to the actual numbers in Figure 5a, which suggests that the model is accurate in representing the real disease dynamics. Peak values of 4 regions including Asia, European Union, North America, and Latin America occur simultaneously around July 1st, leading to extremely high demands for critical medical supplies and severe global production disruptions, which poses extreme challenges in pandemic containment. Figure 5c shows the simulation results for the ODS strategy. In this case, infections are notably reduced due to the dynamic supply chain transformation achieved by NPC. Figure 5d shows simulation results for the OCN strategy. Using this strategy, it is observed that infections are reduced further and pandemic almost fades in July. Note that peaks of regional infections are staggered in both ODS and OCN strategies. Both these optimization-based containment strategies lead to a spatiotemporal separation of infection peaks to split stresses in supply chain. Moreover, these containment strategies also ensure that at least one of major HHS providers is available to support other regions in each period of time, thus preventing an intense growth of the disease.

**Figure 6:**
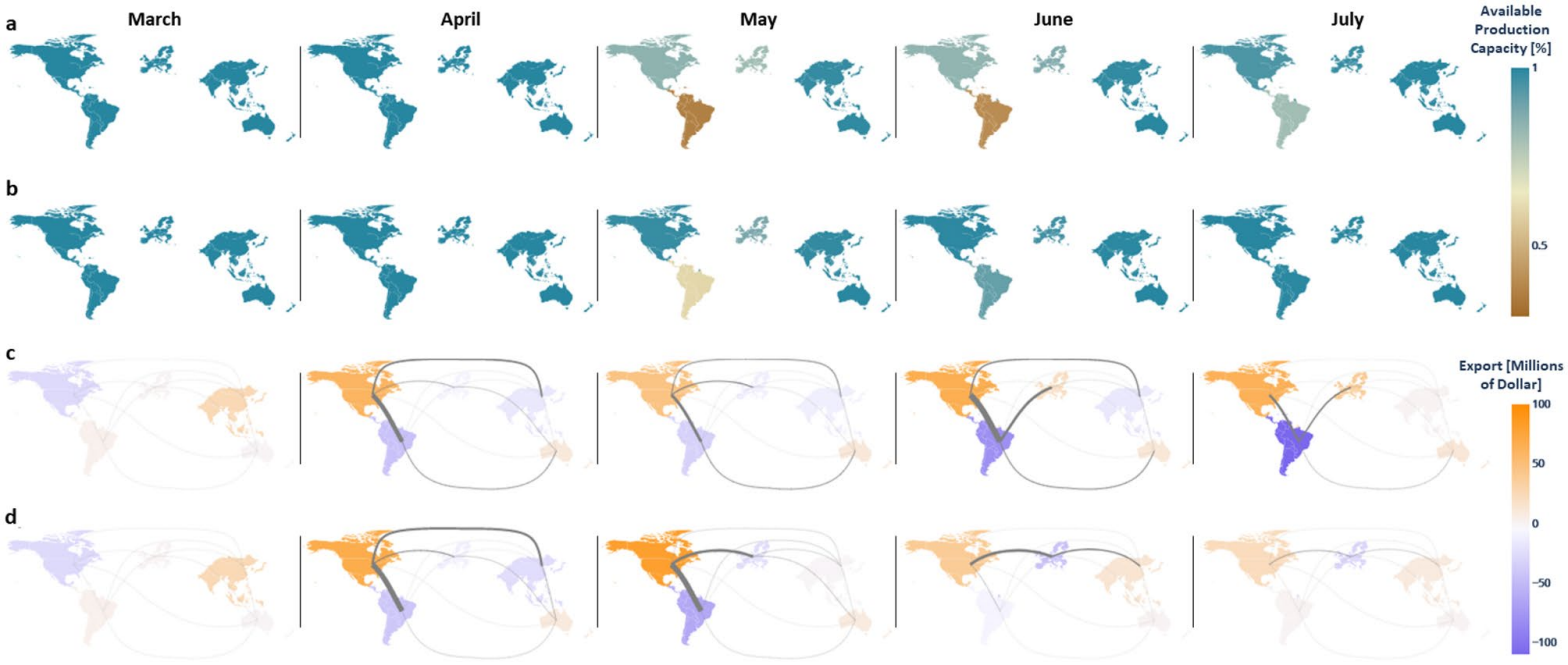
Production disruption and trade decisions of different containment strategies. **(a)** The dynamics of available production capacity by region in the ODS strategy. The color bar represents the percentage of available production capacity of each region. **(b)** The dynamics of available production capacity in the OCN scenario. **(c)** The total import and export decisions of each month in the ODS strategy; The strength of connections of regions marked by the width of the corresponding lines. **(d)** The total import and export decisions of each month in the OCN strategy; the strength of connections of regions marked by the width of the corresponding lines.

Figure 6a summarizes the loss of production capacity of each region in the ODS strategy. Without considering positive feedback, North America and European Union suffer capacity losses in May (15.75% and 19.33%) and June (16.57% and 13.44%). As a comparison, production disruptions for European Union and North America are reduced to 2.11% and 12.15% in May and 0.9% and 3.86% in June respectively in the OCN strategy (Fig. 6b). The better recovery of production capacity results in more HHS resources that can be produced and distributed to other regions for disease control, thus leading to a quick production recovery, especially for regions with stronger industrial capabilities. Note that Latin America suffers a higher production disruption compared to other regions. Lack of capacity of HHS production makes Latin America unable to react to surges in HHS demands under a health emergency, which highlights the need for inter-regional collaboration.

Figure 6c,d show the trade history of the world’s supply chain corresponding to ODS and OCN strategies, respectively. Results from March 2020 are used as benchmark, representing inter-region collaboration simulated in the pre-pandemic strategy. Much stronger collaboration among regions is observed after April when NPC starts. Although Oceania and Latin America both lack the capacity to produce HHS resources, infections in Oceania are delayed, which reserves the production capacity in health care resource preparation ahead of time to contain the disease growth. In contrast, low capacity and high existing infections make Latin America rely on imports from other regions for disease control. The comparison shows that managerial decisions need to be made according to regional production capacity as well as the connectivity to other regions.

Although both containment strategies suggest similar managerial decisions in April, ODS containment strategy does not account for the positive feedback, and thus inaccurately forecasts changes in disease trends from historical medical supplies data. Due to positive feedback, a small difference in predictions may evolve into a distinct outcome for the disease. Asia and European Union receive 50% and 42% more HHS supplies from North America and Oceania in the OCN strategy. Although the importance of additional supplies might not be immediately recognised, it makes a significant long-term impact on containing the disease if applied at the early stages of pandemic. For example, Asia is the import region in the first two months. Increased imports in April and May slow down pandemic growth, which shortens production disruptions and reduces the demand from infected individuals and hospitals. As a result, Asia changes to an export region in June. Our coupled model predicts that the disease is significantly reduced in Latin America by the inter-region collaboration although that is the region hardest hit by the disease. Lower amount of infections in Latin American in turn reduce imported infections to other regions, and that slows the spread of disease.

In the ODS strategy, Asia needs to import HHS resources from other regions all the time. Due to limited health care resources, pandemic in Latin America starts to grow and requires more urgent medical aid from other regions, thus resulting in a totally different pandemic situation as shown in Fig. 5. In July, Latin America still needs to order health care resources to satisfy HHS demands from other regions, while these other regions start to export health care resources while experiencing severe pandemic (e.g., export from European Union). The consideration of positive feedback and the transparency of data regarding supplies enhances the OCN containment strategy, leading to a lean management of HHS resources to better control the growth in HHS demands and to limit the spread of disease.

### Simulated past diseases in the current world

**Figure 7:**
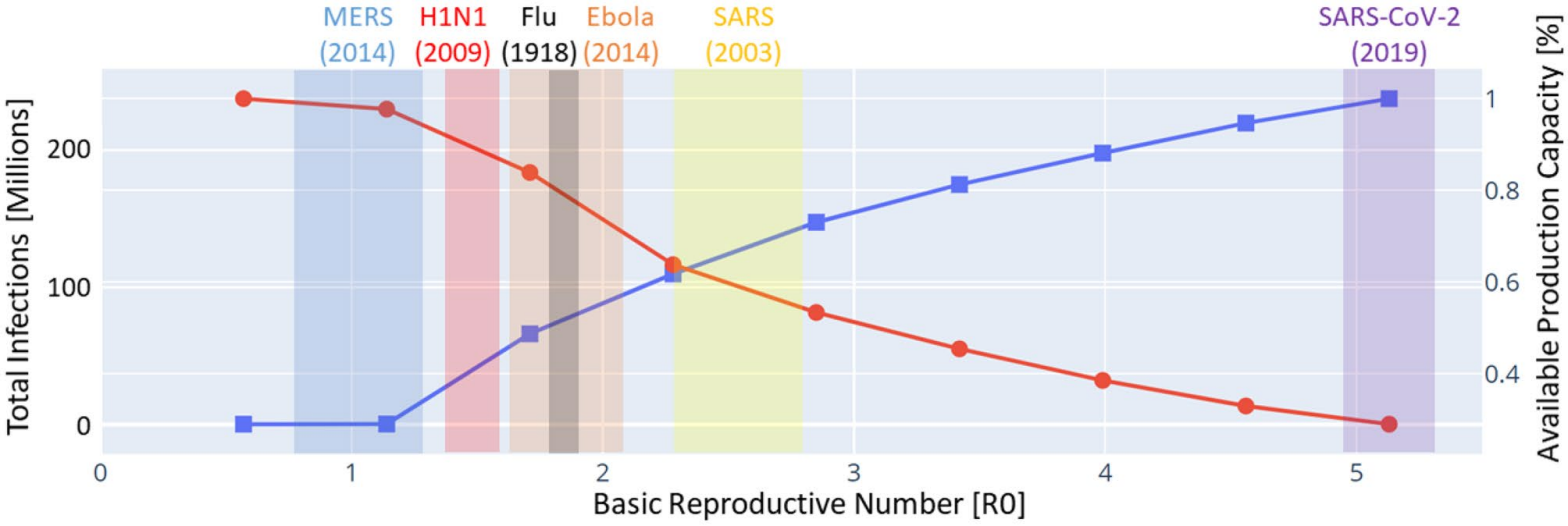
Simulated past infectious diseases in the current world by using the model of coupled networks, which captures time-varying transmission rates changing along with health care resources and NPIs.

The basic reproduction number, $R_0$, is an epidemiologic metric that describes how contagious an infectious disease is before mitigations start^45^. In our study, we consider NPIs (included in the approximated transmission rates) and health care resources (variables to be optimized) as two critical factors that mitigate the spread of disease. We set an initial $R_0$ of SARS-CoV-2 as 5.7 following the previous research^46^ and parameterize the transmission rates by $R_0$. Assuming that supply chain configuration, population size and NPIs remain the same, the expected impact of past infectious diseases, including Spanish Flu (1918)^47^, SARS (2003)^48^, H1N1 (2009)^49^, MERS (2014)^50^ and Ebola (2014)^51^, in the current world situation are demonstrated in Fig. 7.

Compared to other diseases, severe impacts from COVID-19 can be attributed to its extremely high reproduction. Lower available production capacity leads to higher supply chain disruption, which is expected to decrease with increasing $R_0$, as shown in Fig. 7. The number of infected people significantly increases with $R_0$ larger than 1, and an epidemic could ensue. With modeling positive feedback in the coupled model and implementation of real-time NPC, our results indicate that a pandemic breakout occurs with a higher $R_0$ value, 1.14, and the number of infections grows almost linearly with increasing $R_0$. Feedback in our model alleviated the shortage of health care resources mitigating the spread of disease with reduced reproduction number (i.e., $R_t<R_0$). In our model, NPC can effectively leverage the dependency between health care resources and disease control leading to an improved containment strategy.

## Discussion

COVID-19 is neither the first nor the last pandemic that humanity will face. Risks of further pandemic outbreaks of other pathogens exist^4,52^. Until now, most research has focused on the economic impact of public health interventions^53,54^ or on improving the accuracy of disease prediction^55,56^, not the feedback between pandemic growth and supply chain disruption. Supply disruption not only slows down governments’ ability to respond, but also allows the disease to spread by limiting health care resources, which in turn increases demand. Our model views pandemic growth and supply chain disruption as mutually reinforcing pressures. In our model, supply chain and pandemic networks are coupled to analyze the mutual influence in HHS resource shortages, disease dynamics, and production disruption. The NPC algorithm provides agile and time sensitive containment strategies that control the disease by optimizing supply chain managerial decisions, which enhances availability of HHS resources while accounting for trade-offs between disease growth and economic loss.

Our discovery based on real-world data uses the status quo as benchmark to highlight the effectiveness of different containment strategies in what-if scenarios. The existing diversity in economy and public health status in different regions of the world are modelled to address the need for customization of disease containment and inter-region collaboration strategies. The significance of our analysis may be observed by contrasting results of Fig. 5. Foreseeing the positive feedback between supply chains and disease dynamics enhances our understanding of pandemic growth and supply chain disruption, leading to an agile and lean resource allocation strategy that effectively decelerates the spread of the disease and facilitates production recovery.

Shortage of key equipment and materials in health care delivery has been a significant challenge since the beginning of pandemic. Researchers acknowledge that ramping-up HHS resource production may not be as simple as raising the production capacity, but requires the coordination of raw materials^12,15^. In addition, the recent surge in HHS demands was found to stress supply chain networks and deepen disruptions^6,57^. The model we propose not only identifies bottlenecks in ramping up HHS production, but also indicates the minimal sector impacts. Although these discoveries do not reflect all the underlying causes of supply chain disruption, they significantly advance our ability to manage the situation by informing decision makers about vulnerable sectors that have to be coordinated and reinforced during an outbreak.

Given increased HHS production, enhanced inter-region collaboration at the start of pandemic would have shortened the pandemic period. Regional containment strategies are customized automatically in the model according to risk of infection, industrial structure and production capacity, leading to distinct roles of regions in a health emergency. Results highlight the need to strategically distribute the HHS stockpiles differently by region in order to prevent future resource shortages. The active control model of the supply chain network tends to distribute resources to regions with high HHS production capacity to prevent those regions from being disrupted due to lockdown policies caused by pandemic, thus improving long-term HHS production supplies.

Comparing results of OCN and ODS strategies, impacts of modeling positive feedback of disease dynamics and supply chain management was highlighted. The subtle difference in predicting disease dynamics and demands resulted in small shifts in managerial decisions at the early stage of pandemic. These subtle shifts led to a substantial divergence in long-term performance due to reinforcing pressures between disease growth and supply chain disruption. For instance, ignoring positive feedback values makes HHS export countries overestimate HHS demands thus being conservative in exporting, which in turn reduces the amount of HHS resources other regions receive. Increase in HHS shortages accelerates growth of disease, which in turn demands more HHS in the future, leading to a butterfly effect^58^, where a small change in managerial decisions for a coupled nonlinear system can result in large differences in outcomes.

In the study of COVID-19, we quantitatively assessed the impacts of cross-sectoral coordination and agility in containing the outbreak. We explored different containment strategies for critical medical supplies to seek the best managerial outcome in terms of minimizing both the number of fatalities as well as the losses in meeting demands. In our model, managerial decisions are customized based on the ability of different world regions to produce required resources and the risk of infection in terms of transmission rate. Such customized decisions could lead to time-sensitive strategies, illustrating the importance of agility in prediction, lean resource management, and collaboration in pandemic control and economic recovery. Results of this study highlight the importance of cross-sectoral coordination and information transparency between manufacturers across the world to contain a pandemic.

In summary, we proposed a model to analyze the interactive effects of supply chain disruption and infectious disease dynamics using coupled production and disease networks built on global data. We explored different containment strategies for critical medical supplies to seek the best managerial outcome in terms of minimizing both the number of fatalities as well as the losses in meeting demands. Results show that applying active network control strategies leads to time-sensitive strategies, illustrating the importance of agility in prediction, lean resource management, and collaboration in pandemic control and economic recovery. In addition, results of this study highlight the importance of cross-sectoral coordination and information transparency between manufacturers across the world to contain a pandemic. Our model of feedback between supply chain disruption and disease dynamics can be used to quantify the impacts of new technologies, including artificial intelligence^59^, industrial IoTs^60^ and advanced manufacturing technologies^61^ in containing the spread of disease. Thus, the proposed model can be used as a valuable tool to identify the bottlenecks of the supply chain and public health systems in future pandemics.

## Methods

### Coupled supply chain and disease networks

Each region is modeled as a patch with a disease model for simulating the disease dynamics and a production-inventory model for production scheduling and planning. In particular, we use an SEIRHD model of the disease with six compartments^62^, including susceptible (S) reflecting the part of the population that could be potentially subjected to the infection, exposed (E) representing the fraction of the population that has been infected but is not infective yet, infected (I) representing the infective population after the latent period, hospitalized (H) representing the fraction of infected individuals who need hospitalization, recovered (R) representing the population that has successfully recovered from the infection, and dead (D) representing fatalities due to the disease. Production and inventory model of region *i* is described by two states at each time *t*: (1) inventory level of type *k* resource, $V_{i,k}(t)$; (2) backlogged demands for type *k* resource, $U_{i,k}(t)$. We denote type *h* resource as HHS resources. Thus, $V_{i,h}(t), U_{i,h}(t)$ are the inventory level and demand for HHS resources in region *i*. Regional managerial decisions in region *i*, including production of type *k* resource, $w_{i,k}(t)$ and distribution to the public $o_{i,h}(t)$, are decisions to be optimized in local inventory production planning. Resource production follows the bill of materials $M_{k',k}$, which specifies the amount of type $k$ resource required to produce a type $k'$ resource. Parameters in production and inventory model are estimated by using the GTAP datasets.

To model connectivity in the system and the transmission between regions, the infection in region *i* is affected by the number of infected individuals in other regions (due to interrelations such as travel), which creates a multi-patch disease network. We use $\beta _{i,j}$ as the rate at which susceptibles in region *i* are infected by infected individuals from region *j*. We assume that infections can be passed between any pair of regions, albeit possibly via intermediate regions. We assume that $\beta _{i,j} \ll \beta _i$ to reflect the fact that between-patch transmission parameters are significantly smaller compared to within-patch transmission parameters. Cross-region trade and supplies connect regional inventories as a global production and supply chain network involving region-wise trade decisions, i.e. $o^{i',i}_h(t)$ for importing resources of type *k* from region $i'$ to *i*. Summarizing above descriptions, we model dynamics of disease, inventory and demand in region *i* using following set of equations:

1 $$\begin{aligned} S_i(t+1)= S_i(t)-S_i(t)\sum _{j}{\frac{\beta _{i,j}(t)I_j(t)}{N_j}}, \end{aligned}$$

2 $$\begin{aligned} E_i(t+1)= E_i(t) + S_i(t)\sum _{j}{\frac{\beta _{i,j}(t)I_j(t)}{N_j}}-\gamma _{EI}E_i(t), \end{aligned}$$

3 $$\begin{aligned} I_i(t+1)= I_i(t) + \gamma _{EI}E_i(t)-(\gamma _{IH}+\gamma _{IR})I_i(t), \end{aligned}$$

4 $$\begin{aligned} H_i(t+1)= H_i(t) + \gamma _{IH}I_i(t)-(\gamma _{HR}+\gamma _{HD})H_i(t), \end{aligned}$$

5 $$\begin{aligned} R_i(t+1)= R_i(t) + \gamma _{IR}I_i(t)+\gamma _{HR}H_i(t), \end{aligned}$$

6 $$\begin{aligned} D_i(t+1)= D_i(t) + \gamma _{HD}H_i(t), \end{aligned}$$

7 $$\begin{aligned} V_{i,k}(t+1)= V_{i,k}(t) + \sum _{i'}{o_{i',i,k}(t) - o_{i, i',k}(t)} - \sum _{k'}M_{k',k}w_{i,k'}(t) + w_{i,k}(t) - o_{i,k}(t), \end{aligned}$$

8 $$\begin{aligned} U_{i,k}(t+1)= U_{i,k}(t) + \delta U_{i,k}(t) - o_{i,k}(t), \end{aligned}$$

where, $N_i$ is the size of population of region *i*. $\gamma _{IH}=\delta _H/\tau _I$, $\gamma _{IR}=(1-\delta _H)/\tau _I$, $\gamma _{HD}=\delta _D/\tau _H$, and $\gamma _{HR}=(1-\delta _D)/\tau _H$, where *c* is the percentage of infected individuals hospitalized, and $\delta _D$ is the fatality rate. $\tau _I$ and $\tau _H$ represent the average infectious period and average hospital stay in days, respectively. In our analysis, we choose $\gamma _{EI}=1/5.2$ days, $\tau _{I}=4.6$ days, and $\tau _{H}=10$ days according to values reported in previous studies^63^. We fit the model to data of COVID-19 to find the coefficients $\delta _{Di}, \delta _{Hi}$ and $\beta _{i,j}$ at each time instant. The proposed modeling framework is generalized for different types, intensities, and duration of interventions to be implemented in each region, and thereby illustrates how these interventions impact disease dynamics and resulting number of infections and fatalities through time. In particular, we consider non-pharmaceutical interventions (NPIs) (e.g., social distancing) and the shortage of HHS resources for a set duration applied as a scaling of transmission rates for all infected individuals.

### Coupling functions

#### Disease-dependent capacity function

Studies have confirmed the dependence of optimal lockdown policy on the disease fraction in the population^28^, which links pandemic severity to production capacity. Assuming that optimal lockdown policy is adopted, available production becomes a function of existing disease situation, i.e., the percentage of infected and hospitalized. These simplifications allow us to describe a real-time evolving supply chain network, together with disease spreading on it, to evaluate the amount of production capacity available during pandemic, namely

9 $$\begin{aligned} w_{i,k}(t)\le W_{i,k} = e^{-\gamma _w\frac{I_i(t)}{N_i}}\bar{W}_{i,k}, \end{aligned}$$

where $\bar{W}_{i,k}$ is the pre-pandemic production capacity and $\gamma _w$ is a scaling parameter designed such that only 1% production capacity remains available when the percentage of active infections reaches 1% of the total population.

#### Disease-dependent demand function

There is a broad range of estimates of critical medical supplies required to care for COVID-19 patients, which vary depending on the number, speed, and severity of infections^15,24,64^. Thus, we created a function to characterize how governments and households issue orders to their manufacturers under pandemic. Demands are separated into commercial resources, $\delta U_{i, k}, \forall k\ne h$ and HHS resources, $\delta U_{i,h}$. Additional HHS resources are considered to be dependent on the severity of pandemic and correlated to the number of existing infected individuals $I_i(t)$ and hospitalized individuals $H_i(t)$. The product demands of other sectors remain the same as pre-pandemic, namely

10 $$\begin{aligned} \delta U_{i,h}(t)=\, &  \gamma _II_i(t) + \gamma _HH_i(t) + a_{i,h}(t), \end{aligned}$$

11 $$\begin{aligned} \delta U_{i,k}(t)=\, &  a_{i,k}(t), \qquad \forall k \ne h, \end{aligned}$$

where $\gamma _I$ and $\gamma _H$ measure expenditures needed to treat a susceptible individual and an infected individual on a daily basis (we assume $\gamma _I = 0.0001, \gamma _H = 0.002$), which are roughly estimated based on the direct medical costs of MERS coronavirus^65^. $a_{i,k}(t)$ is daily consumption of resources of type *k* during the pre-pandemic period.

#### Shortage-dependent transmission function

To model the transmission rate, we consider three elements for each region *i*, including a base transmission rate $\beta ^i_0(t)$ representing the transmission rate without any NPI measures, a health care coefficient $c_{H,i}(t)$ representing reduction in the transmission rate due to proper usage of HHS resources, and an NPI coefficient $c_{N,i}(t)$ representing the reduction in transmission rate due to change in economic activity and NPI such as social distancing and curfew. We define $c_{H,i}(t) = 1 - \frac{2(1-P_l)}{1+e^{\frac{10^6U_{i,h}(t)}{N_j}}}$, where $P_l$ is the minimum protection measure with zero health care stocks ($P_l=0.5$, reflecting 50% effectiveness of HHS products if used properly^30,66^). Based on these coefficients, the disease transmission rate $\beta _{i}(t)$ of region *i* is expressed as

12 $$\begin{aligned} \beta _{i}(t)= \beta _{i,0}(t) c_{N,i}(t) c_{H,i}(t). \end{aligned}$$

#### Shortage-dependent fatality function

This function models the feedback between the shortage of health care resources and the fatality rate $\delta _D$. Denote the transition rates from hospitalized to recovered and to dead as a function of the fatality rate $\delta _D$, namely, $\gamma _{HR} = (1 - \delta _D) /\tau _h$ and $\gamma _{HD} = \delta _D/ \tau _h$. We define $\delta _{D0}$ as the base fatality rate of hospitalized individuals assuming sufficient health care resources. $\delta _{D1}$ measures the increment of the fatality rate under the shortage of health care resources. Under this assumption, we model the fatality rate as a time-varying variable depending on the shortage in health care resources, namely

13 $$\begin{aligned} \delta _D(t) = \delta _{D0} + c_{h}(t) \delta _{D1}, \end{aligned}$$

where $c_{h}$ is 0 when health care resources are sufficient, and is 1 when there is no stock of health care resource. All parameters are calculated directly from real-world data or from parameter fitting unless specified.

### Parameter fitting

We use the data reported by Johns Hopkins University Center^38^ from January 22, 2020 to September 24, 2020 as our dataset for parameter fitting of coupled model. Data includes time-series of daily updates on the new infected cases, fatalities and recovered cases. The objective is to identify parameters of the compartmental model in such a way that the simulated data matches the data as much as possible. The simulated data are obtained by numerically solving the model in Eqs. (1)–(6) using an integration algorithm.

A two-step fitting process is performed to obtain the best parameters for the system of disease dynamics consisting of six regions. In the first step, we considered the disease in each region independently without considering the cross-coupling terms ($\beta _{ij},i \ne j$) in the dynamics. To accomplish the parameter identification, we solved a nonlinear constrained least-squares problem for each region to minimize the cost function between the model prediction and the data. We devised the cost function as a sum of the mean square error for daily infected and fatalities. We did not include the recovered individuals in the cost function since the definition of recovery in the equations (i.e., not infectious) and those reported in data (i.e., cured) might not be consistent. The initial number of infected and recovered cases for the SEIHRD model was considered. Also, due to lack of information regarding the initial number of exposed individuals ($E_0$), it was assumed to be five times of the initial number of infected individuals. To capture rapidly changing social scenarios particularly during the initial period of the COVID-19 spread, we split the integration interval into sub-intervals of 20 days, and the best parameters ($\beta _{i}, \delta _{Hi}, \delta _{Di}$) were found at each sub-interval (the parameters are assumed to be constant in each sub-interval).

**Figure 8:**
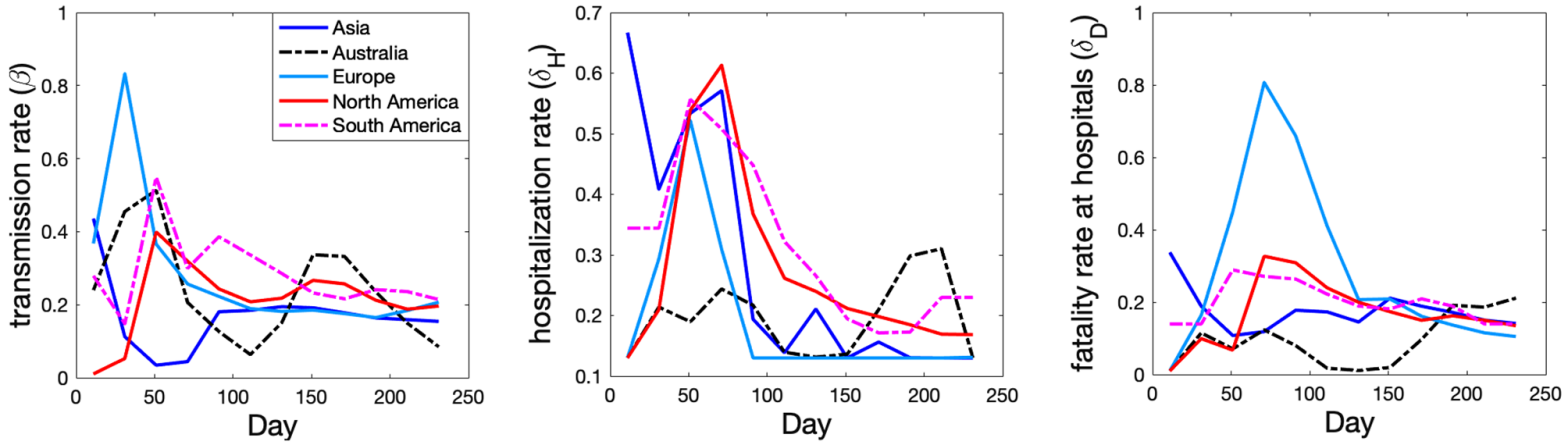
Disease parameters obtained by fitting the coupled equations in coupled model to COVID-19 data starting from January 22, 2020 to September 24, 2020.

Next, we use the fitted parameters as an initial guess to fit the coupled equations (1) and (2) simultaneously for all regions considering the cross-coupling terms. The disease in each region is connected to that of other regions by cross-transmission terms, $\beta _{i,j},i \ne j$. To identify the best parameters for the coupled model, we fix values of $c_{i}$ and $d_{i}$ and search for the best transmission matrix *G* that fits the data. We define the transmission matrix *G* as $G_{i,j}(t)=\epsilon p_{i,j}(t) \beta _{i}(t), i \ne j$, $G_{i,j}(t)=\beta _{i}(t),i=j$, where $p_{i,j}$ is a coefficient proportional to the number of daily travels from region *j* to region *i* adjusted by the change in the international air travels reported by International Air Transport Association (IATA) since starting pandemic^67^, and $\epsilon$ is a fixed small number ($\epsilon \ll 1$) reflecting that cross-coupling has considerably smaller effect on the disease transmission compared to the internal transmission. We find the best $\beta _{i}$ of each time *i* by using a moving window. It is observed that the suggested model and parameter estimation approach fit the data with a reasonable accuracy. Model parameters for each region is shown in Fig. 8.

### Network proactive control

Coupled model of disease and supply chain networks can be used to predict impacts of managerial decisions and to yield a proactive containment strategy. Due to the nonlinearity in the disease dynamics and coupling functions, linear approximation with Tayler series is implemented. Parameters of the coupled model are updated in each time point to enhance the accuracy of the approximation. For a specific time point *t*, linearized disease dynamics, Eqs. (1)–(6), of time *t* follow

14 $$\begin{aligned} S_i(t +1)= &  \mu _0 + (1+\mu ^i_1) S_{i}(t ) + \sum _j \mu ^{i,j}_2 I_{j}(t ) + \sum _j \mu ^{i,j}_3 U_{j,k}(t ) +\epsilon _1^i, \end{aligned}$$

15 $$\begin{aligned} E_i(t +1)= &  -\mu _0 -\mu ^i_1 S_{i}(t ) - \sum _j \mu ^{i,j}_2 I_{j}(t ) - \sum _j \mu ^{i,j}_3 U_{j,k}(t ) + (1-\gamma _{EI})E_i(t ) - \epsilon _1^i, \end{aligned}$$

16 $$\begin{aligned} I_i(t +1)= &  I_i(t) + \gamma _{EI}E_i(t)-(\gamma _{IH}+\gamma _{IR})I_i(t), \end{aligned}$$

17 $$\begin{aligned} H_i(t +1)=  &  H_i(t ) + \gamma _{IH}I_i(t )-\tau _H H_i(t ), \end{aligned}$$

18 $$\begin{aligned} R_i(t +1)= &  R_i(t ) + \gamma _{IR}I_i(t ) + \gamma _{HR}(t) H_i(t ) + \mu ^i_4 U_{i,h}(t ) + \epsilon _2^i, \end{aligned}$$

19 $$\begin{aligned} D_i(t +1)=  &  D_i(t ) + \gamma _{HD}(t)H_i(t ) - \mu ^i_4 U_{i,h}(t ) - \epsilon _2^i, \end{aligned}$$

where

20 $$\begin{aligned} \mu ^i_1=  &  \frac{\partial S_i(t+1)}{\partial S_i(t)}\biggr |_{S_i(t), E_i(t), U_{i,h}(t), I_j(t)} = -\sum _j {\frac{\beta _{i,j}(\theta ^j(t), U_{i,h}(t) )I_j(t)}{N_j}}, \end{aligned}$$

21 $$\begin{aligned} \mu ^{i,j}_2=  &  \frac{\partial S_i(t+1)}{\partial I_j(t)}\biggr |_{S_i(t), E_i(t), U_{i,h}(t), I_j(t)} = - {\frac{\beta _{i,j}(\theta ^j(t), U_{i,h}(t) )S_j(t)}{N_j}}, \end{aligned}$$

22 $$\begin{aligned} \mu ^{i,j}_3=  &  \frac{\partial S_i(t+1)}{\partial U_{i,h}(t)}\biggr |_{S_i(t), E_i(t), U_{i,h}(t), I_j(t)} = \frac{2S_i(t)I_j(t)c_{N,i}(t)\beta _0(P_l-1)}{N_j(P_l+1)}\frac{\frac{10^6}{N_i}e^{\frac{10^6}{N_i}U_{j,h}(t)}}{(1+e^{\frac{10^6}{N_i}U_{j,h}(t)})^2}, \end{aligned}$$

23 $$\begin{aligned} \mu ^{i}_4=  &  \frac{\partial R_i(t+1)}{\partial U_{i,h}(t)}\biggr |_{S_i(t), E_i(t), U_{i,h}(t), I_j(t)} = H_i(t)\tau _H\delta _{D1}\frac{2(P_l-1)}{(1+P_l)}\frac{\frac{10^6}{N_i}e^{\frac{10^6}{N_i}U_{i,h}(t)}}{(1+e^{\frac{10^6}{N_i}U_{i,h}(t)})^2}, \end{aligned}$$

24 $$\begin{aligned} \epsilon _1^i= &  - [\mu ^i_1, \mu ^{i,j}_2, \mu ^{i,j}_3][S_{i}(t), I_{j}(t), U_{j,h}(t)]^T, \end{aligned}$$

25 $$\begin{aligned} \epsilon _2^i=  &  -\mu ^{i}_4 U_{i,h}(t). \end{aligned}$$

The disease-dependent capacity function in Eq. (9) is also linearized to obtain

26 $$\begin{aligned} w_{i,k}(t ) \le - \frac{e^{-\frac{\gamma _{w} I_{i}(t)}{N_i}}}{N_i}I_i(t ) + \left( 1+\frac{\gamma _{w} I_{i}(t)}{N_i}\right) e^{-\frac{\gamma _w I_i(t)}{N_i}}\bar{W}_{i,k}. \end{aligned}$$

There is no action that is exerted into the disease directly, but the changes in the supply chain propagate to the disease dynamics by affecting remaining demands of HHS resources, $U_{i,h}(t)$ in Eqs. (14)–(19). Analysis of coupled model enables managerial decisions to be made with aware of changes in both disease and supply chain to trade-off the objectives in disease growth and economic losses. Containment strategies are formulated by the following mathematical model, aiming to minimize the total fatalities, economic losses, and managerial cost

27 $$ \begin{aligned}   \mathop {\min }\limits_{{w_{{i,k}} ,o_{{i,k}}, o_{{i,i^{\prime},k}} }} {\kern 1pt}  &  \sum\limits_{{\tau  = t + 1}}^{{\tau  = t + t_{p} }}\sum\limits_{i} \left[{D_{i}}(\tau) +\sum \limits_{k} [{c_{u}}{U_{i,k}}(\tau)+c_{p,k}w_{i,k}(\tau)+\sum\limits_{{i^{\prime} \ne i}} {c_{l,k}}o_{{i,i^{\prime},k}} (\tau )]\right] \\    {\text{s.t.}}\quad \quad  & (a)\;o_{{i,i^{\prime},k}} (\tau ),w_{{i,k}} (\tau ),o_{{i,k}} (\tau ) \ge 0,\quad \forall i,i^{\prime},k,\tau  \\     & (b)\;V_{{i,k}} (\tau ),U_{{i,k}} (\tau ) \ge 0,\quad \forall i,k,\tau  \in [t,t + t_{p} ] \\     & (c)\;w_{{i,k}} (\tau ) \le  - \frac{{e^{{ - \frac{{\gamma _{w} I_{i} (\tau)}}{{N_{i} }}}} }}{{N_{i} }}I_{i} (\tau ) + (1 + \frac{{\gamma _{w} I_{i} (\tau)}}{{N_{i} }})e^{{ - \frac{{\gamma _{w} I_{i} (\tau)}}{{N_{i} }}}} \bar{W}_{{i,k}} ,\quad \forall i,k,\tau  \in [t,t + t_{p} ] \\     & (d)\;V_{{i,k}} (\tau ) \le \bar{V}_{{i,k}} ,\quad \forall i,k,\tau  \in [t,t + t_{p} ], \\  \end{aligned}  $$

where $t_p$ is the planning horizon, which is 14 days in this study, $c_u$ is the weight of economic losses compared to total fatalities in the planning horizon, $c_{p,k}$ and $c_{l,k}$ are production and trade costs to obtain a resource of type *k*, where $c_{p,k} < c_{l,k}$, $\bar{V}_{i,k}$ and $\bar{W}_{i,k}$ specify the maximum production capacity and inventory capacity. Constraint (a) ensures that all supply chain management decisions are non-negative; (b) indicates that the amount of inventory stocks are non-negative; (c) ensures that production decisions of a certain area are always constrained by the available capacity $\bar{W}_{i,k}$; (d) ensures that the inventory stocks are constrained by a regional inventory threshold $\bar{V}_{i,k}$. The model is solved by the *Cplex* package^68^. Managerial decisions are imported to the coupled model described by Eqs.(1) to (8) to simulate the changes in demand, inventory and disease. Managerial decisions are updated in real-time (i.e., daily) to enhance the sensitivity to uncertainties in pandemic and supply chain.

## Data Availability

*Global trade dataset* The global trade dataset used to stimulate the presented results are licensed by the Global Trade Analysis Project at the Center for Global Trade Analysis in Purdue University’s Department of Agricultural Economics. The analyses performed in this paper are based on version 10 of the dataset. Due to the restriction in the licensing agreement with GTAP, the authors have no right to disclose the original dataset publicly. *COVID-19 dataset* We use the data reported by Johns Hopkins University Center as our dataset for model fitting. All data and methods needed to reproduce the results in the paper are provided in the paper or as supplementary materials. This dataset is publicly available at (https://raw.githubusercontent.com/datasets/covid-19/master/data/countries-aggregated.csv)

## Code availability

The code will be available upon the request.
